# *Giardia duodenalis* in Captive Tigers (*Panthera tigris*), Palawan Bearcats (*Arctictis binturong whitei*) and Asian Palm Civet (*Paradoxurus hermaphroditus*) at a Wildlife Facility in Manila, Philippines

**Published:** 2017

**Authors:** Nick Angelo P. VELANTE, Rey B. ORONAN, Marco F. REYES, Billy P. DIVINA

**Affiliations:** 1. Cargill Philippines Inc, Pulilan, Bulacan, Philippines; 2. Dept. of Veterinary Clinical Sciences, College of Veterinary Medicine, University of the Philippines Los Baños, Laguna, Philippines; 3. Dept. of Veterinary Paraclinical Sciences, College of Veterinary Medicine, University of the Philippines Los Baños, Laguna, Philippines

**Keywords:** *Giardia duodenalis*, Palawan bearcat, Palm civet, Tigers

## Abstract

**Background:**

This study was conducted to determine the prevalence of *Giardia duodenalis* in captive animals in a wildlife facility. This is the first study conducted in these animals from the facility.

**Methods:**

Eight captive tigers (*Panthera tigris*), two Palawan bearcats (*Arctictis binturong whitei*) and one Asian Palm Civet (*Paradoxurus hermaphroditus*) currently housed at a wildlife facility in Manila, Philippines were considered in 2012. These animals were apparently healthy with no signs of disease during the study. Sample collection was done twice at two months interval where freshly voided fecal samples were grossly examined, characterized and preserved in Sodium Acetate Formalin (SAF). The samples were used to determine the presence of *G. duodenalis* using modified flotation-sedimentation and commercially available immuno-chromatographic assay test kit.

**Results:**

All fecal samples tested were negative for the presence of *G. duodenalis* trophozoites, and cysts using the former. Furthermore, none of the samples tested positive for and *G. duodenalis* antigen using immune-chromatographic assay.

**Conclusion:**

There is no existing infection of *G. duodenalis* among captive tigers, Palawan Bearcats and Asian palm civet housed at the wildlife facility.

## Introduction

*Giardia* sp. are single-celled, intestinal parasitic flagellates found in the intestines of a wide range of domestic and mammalian hosts including dogs, cats, deer mouse, ground squirrel, chinchilla, swine, pocket mouse, ox and guinea pig (1–3). The organism consists of six species, distinguished based on the morphology and the ultra-structure of its trophozoites (2).

Among the members of the genus *Giardia*, *G. duodenalis* is the only species found in humans originally regarded as a commensal organism of vertebrate mammals (4). The presence of the *G. duodenalis* usually presents subclinical infections (4) to severe diarrhea and chronic gastrointestinal disease. Less common symptoms are nausea, vomiting, appetite and weight loss (5). Because of these signs, determination of definitive diagnosis is difficult (4).

Giardiasis is a common and widespread intestinal protozoal disease (2). Both humans and animals may become infected either by direct fecal ingestion or by the ingestion of contaminated food & water (6, 7). Freshly passed cysts are immediately infective and cysts are hardy and can survive several months in cold environment. The ingestion of as few as ten *Giardia* cysts is enough to cause infection. The condition is zoonotic and is a cause of major public and veterinary health concern in both developing and developed countries worldwide (6, 8) with infection ranging from five to 70% (5, 9).

*G. duodenalis* has been found in the small intestines of healthy individuals in endemic areas (4). *Giardia* infection is the most common intestinal parasitic disease-affecting humans in the US (9). In the Philippines, the prevalence of giardiasis is 2%, with the highest frequency reported from Mindanao. Furthermore, it has also been more frequent in younger individuals compared to adults (5, 10).

Although giardiasis in animals has been established in the wild, there are very few studies conducted on its prevalence on wild animals. In the US, trophozoites have been isolated in wild dogs, acts, deer and field mice (11, 12). However, the last study to detect *Giardia* sp. in the country was conducted and published back in 1930’s (13). No other study has been conducted to determine the giardiasis in captive, rescued or rehabilitated animals in wildlife facilities in the country.

This study aimed to determine the prevalence of *G. duodenalis* in captive tigers (*Panthera tigris*), Palawan bearcats (*Arctictis binturong whitei*) and Asian palm civet (*Paradoxus hermaphroditus*) housed in a wildlife facility in Manila, Philippines. The results of the study will confirm the presence of the protozoal parasite. Furthermore, the study will provide a clinical background of these animals to ensure management and prevent future infections. Lastly, the study hopes to pave the way for further studies and future researches on the epidemiology and zoonotic risks of *G. duodenalis* in a wildlife facility, its animals, and staff alike.

## Materials and Methods

The procedures described in this study were submitted and approved by the College of Veterinary Medicine Institutional Animal Care and Use Committee, Protocol No.: 2011-22 of the University of the Philippines Los Baños, Laguna, Philippines.

Eleven animals in 2012, of both sexes and varying ages, belonging to order Carnivora under the suborder Feliformia were considered in this study. Eight were captive tigers (*P. tigris*), two Palawan bearcats (*A. binturong whitei*) and one Asian palm civet (*P. hermaphroditus*) housed in a wildlife facility in Manila, Philippines. The animals were selected primarily due to the proximity of their enclosures to another.

The tigers are given a mixture of carabeef and chicken. A carabao’s leg is also given every 15 days while drinking water is given ad libitum. The Palawan bearcats and Asian palm civet were given fruits and vegetables twice daily. Fecal examination and deworming with ivermectin (Ivomec® 1% injection; Merial) are done biannually and Rabies vaccination annually. All the animals used in this study were apparently healthy and free from infectious diseases at the time of the study.

All the animals were observed in their respective individual enclosures. These enclosures were cleaned daily with water and disinfected quarterly with sodium hypochlorite. Furthermore, the tigers had a communal viewing pen but were placed individually in concrete enclosures until defecation was observed. Once observed, the tigers were released back into the communal pen. The Palawan bearcats and Asian palm civet were observed in their enclosure until defecation was seen.

### Collection of Fecal Sample

Fresh fecal samples were collected immediately after being voided by the animal via direct handling with disposable sterile gloves. The collected samples were grossly observed for any indications of parasitism. The characteristics like fecal consistency, color and texture were examined, compared, recorded and samples were stored in individual 30 ml sterile disposable plastic container labeled properly: animal identification code, age, and sex. The gross characteristics were compared using a seven point fecal scoring system (Nestle Purina® Fecal Scoring Chart) with the following description: Score 1- very hard and dry; Score 2- firm but not hard, pliable, segmented in appearance; Score 3- log-like, little or no segmentation visible, moist surface; Score 4- very moist and soggy with visible distinct log shape; Score 5- very moist but has log shape, losses form when picked up; Score 6- has texture but no defined shape and; Score 7- watery, no texture, occurs as puddles. The fecal samples were sealed and stored under refrigeration (4 to 5 °C) until processing. Sampling was done on twice, at two months interval.

**Fig. 1:**
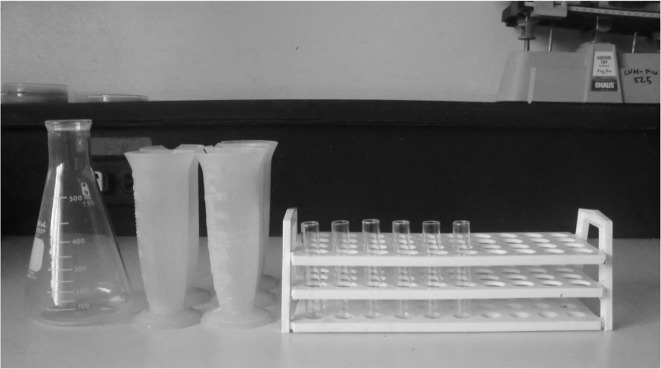
Modified-Flotation Method set up showing 15-mL test tubes, test-tube rack, Erlenmeyer flask and plastic graduated cups

The fecal samples were initially tested using the Modified-Flotation method using 40% concentrated sugar solution, the set-up of which is shown in Fig. 1. Approximately 2 mg of stool sample was handled using a sterile, disposable wooden tongue depressor and preserved in 15ml Sodium Acetate Formalin (SAF). The individual samples were placed in 60ml sterile screw capped vessels containing the SAF solution. The samples were thoroughly mixed and the wooden tongue depressors were discarded after each sample. This was done for all fecal samples.

### Modified Flotation-Sedimentation Method

Prior to testing the samples in SAF fixative were allowed to thaw at room temperature. Approximately 1mL of the preserved fecal sample was transferred to a paper cup using a disposable tongue depressor. Ten milliliters of distilled water was measured using a 15-mL graduated cylinder (Pyrex®) and added to the paper cup with the fecal sample. The mixture was stirred until the fecal material was thoroughly mixed. Using a sieve, the mixture was strained and filtrate was transferred to another paper cup. The filtrate was then placed in a 15-mL test tube for easier decantation. The test tube was allowed to stand for 30 min.

The supernatant was then decanted carefully leaving the sediments undisturbed. The process was repeated until the supernatant became clear and the filtrate was centrifuged at 12000 rpm for three minutes using an IEC HN-SII centrifuge. The suspension after centrifugation was decanted. The test tube containing the sediments was then filled with 40% sugar solution until a meniscus appeared above the lip of the test tube. A 22 × 22 mm cover slip was then placed gently on top of the meniscus where it was allowed to settle for 15–20 min, after which it was lifted straight up and immediately placed on the microscope slide. The slide was placed on the microscope for examination of the entire microscopic field.

### Microscopic Identification

Samples were examined using a binocular compound microscope under 40×, 100× and 400× magnification using the Meander method (14). Presence of parasite cysts and trophozoites were noted and comparison for size, shape, and structure of protozoan observed using standard references were performed.

**Fig. 2:**
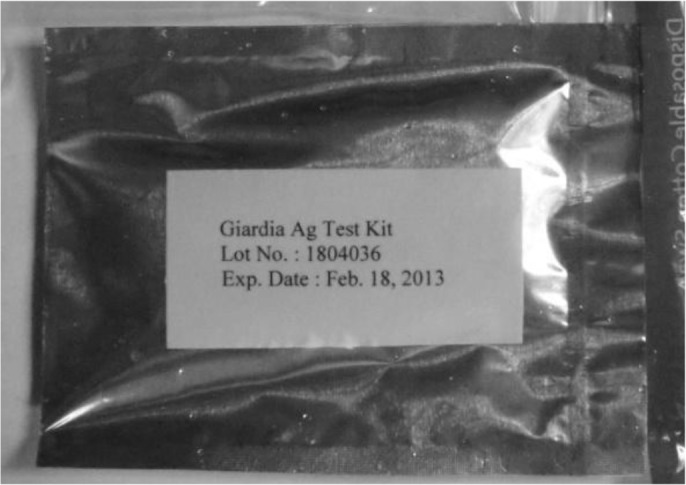
A commercially available immunochromatographic antigen test kit (ImmunoRun® Antigen Detection Kit-BioGal-Labs, Israel) specifically used for the detection of *Giardia duodenalis* antigen from fecal samples.

The rest of the fresh and unpreserved fecal samples were further tested using ImmunoRun® Antigen Detection Kit: *Giardia* (Fig. 2) to confirm the results of the Modified-Flotation method previously performed.

### ImmunoRun® Antigen Detection Kit: Giardia

ImmunoRun® Antigen Detection Kit: *Giardia* (BioGal-Laboratories, Israel) is an immunochromatographic assay kit intended for detection of *G. duodenalis* antigen in dog and cat feces. It is the simplest screening diagnostic method to detect the presence of *G. duodenalis* antigen in fecal samples. It has been validated to give 100% sensitivity and specificity.

The test kit contains 5 individual devices to qualitatively detect CFG-Ag in canine or feline feces. Each device contains 2 main windows: a round window, which is the specimen application well, and a rectangular result window, marked by two letters: ”C” for Control line and “T” for Test line. Both lines are invisible before reaction takes place. The manufacturer’s recommendations and directions were followed in the conduct and interpretation of the antigen kit.

## Results

**Fig. 3:**
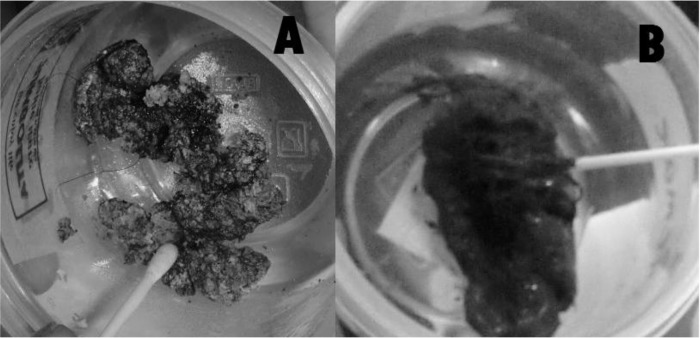
(A) Fecal sample from a Palawan Bearcat scored as 1, with a description of very hard and dry; a (B) Fecal sample from a tiger scored as 3, with a description of being log-like with no visible segmentation and moist surface

Eleven captive animals, composed of eight tigers, two Palawan bearcats, and one Asian Palm civet, from a wildlife facility were used for the study. The fecal samples were grossly assessed and graded using the 7-point fecal scoring system (Nestlé PURINA® Fecal scoring chart). The fecal samples of the Palawan bearcats (Fig. 3A) and the Asian palm civet showed that the sample was hard and dry and was graded with a score of one. On the other hand, the fecal samples from tigers were graded with an average score of 3, described as log-like with little or no segmentation visible. Furthermore, the fecal sample of the tigers was moist (Fig. 3B).

The presence of *Giardia* sp. cysts were assessed using the modified flotation-sedimentation method. Sample collection was done twice at two-month intervals as *Giardia* cysts are intermittently shed. No *Giardia* cysts or trophozoites were identified upon examination of wet mounts derived from the modified flotation-sedimentation method from all samples in both sampling dates. Cysts can usually be observed using a centrifugal flotation (7).

**Fig. 4:**
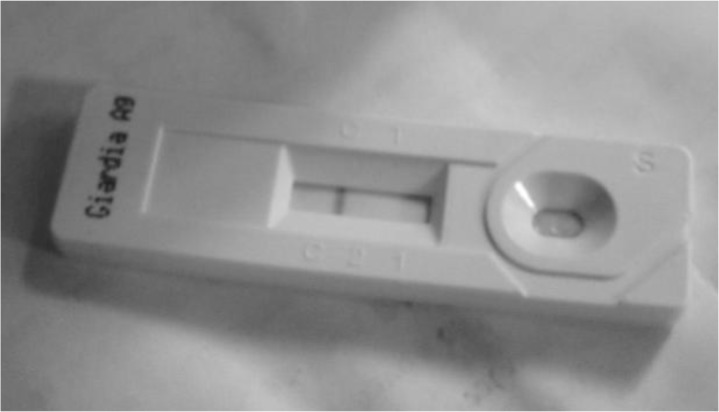
ImmunoRun® Antigen Detection Kit showing negative results as indicated by the single bar that indicated the positive control of the detection kit

Furthermore, it can also be examined and detected using sugar flotation. However, the hypertonic nature of the solution results in the rapid collapse of the wall of the cyst that may appear as small crescent moons (7). However, an *Isospora*-like cyst was observed. Further testing is recommended to identify the organism observed. Because cysts excretion has been shown to be sporadic (2), it may be best to examine samples by both flotation and antigen detection. All fecal samples were then tested using the commercially available immunochromatographic assay test kit (ImmunoRun® Antigen Detection Kit: *Giardia*). All the fecal samples, for both sampling dates, were negative for *G. duodenalis* antigen (Fig. 4)

## Discussion

In 1930, Chu reported the presence of *G. hegneri* isolated in the small intestines of an apparently healthy Philippine civet cat (13). To date, the study is the first to be conducted to determine the prevalence of *G. duodenalis* in captive tigers, Palawan Bearcat and Asian palm civet in a wildlife facility in the Philippines using both Modified flotation-sedimentation method and the commercially available immunochromatographic assay test kit (ImmunoRun® Antigen Detection Kit: *Giardia*). None of the fecal samples tested positive for *Giardia duodenalis*, for both sampling dates. There is no *G. duodenalis* infection among the captive tigers, Palawan Bearcats and Asian Palm Civet housed at the Manila Zoo thus indicating that there is zero prevalence the disease amongst the said animals.

Several factors may have contributed to the results of this study. One factor to be considered is that giardiasis is a self–limiting infection (15), which means that these animals may have been previously infected but may have already eliminated the parasite before they were brought to the facility. Furthermore, the sporadic nature by which the cysts are excreted could have also been a factor. The researchers tried to do pair sampling at two months interval and still, none of the animals demonstrated *G. duodenalis* in the fecal samples. Multiple sampling per animal is performed with a shorter period of interval.

Although *Giardia* cysts can usually be detected in the feces of an infected animal by a centrifugal flotation procedure (7), fecal flotation is not 100% accurate and due to the intermittent shedding of *Giardia* cysts and trophozoites, it is difficult to demonstrate the organism from the procedure. Another factor to consider is that a different species of *Giardia* might be present in the animals. The use of a commercially available immunochromatographic assay test kit (ImmunoRun® Antigen Detection Kit: *Giardia*®) was employed to confirm the results obtained. The kit has a 100% specificity and sensitivity and is specifically prepared to detect *G. duodenalis* antigen*.* An infection from another species of *Giardia* might be present but was not detected by the test kit.

Lastly, the captive animals considered in this study are truly negative for the disease. Further testing using other methods is recommended to not only rule out *Giardia* infection but also as a basis for the health status and prevent possible future infection in these animals.

As a recommendation, all susceptible animals currently housed at this animal facility should be screened for this parasite. Furthermore, screening for *G. duodenalis* should also be performed to all newly acquired, rescued or donated susceptible animals that are brought in the facility. Animals that tested positive should not be mixed with those that tested negative for the disease. A strict quarantine and treatment regimen along with proper biosecurity measures should be set in place to prevent entry and spread of the disease to other susceptible animals and most importantly to humans. More importantly, regardless of being negative for the parasite, strict hygiene, sanitation, and proper staff and guest traffic should be done to prevent possible zoonosis and disease transmission to humans.

## Conclusion

Two different detection techniques were employed in this study and both techniques were unable to detect *G. duodenalis* in the fecal samples obtained from the eight captive tigers, two Palawan Bearcats, and one Asian palm civet. Therefore, the prevalence of *G. duodenalis* for these animals in this wildlife facility is zero percent (0%).
